# How do Individuals Form Their Motivations to Expatriate? A Review and Future Research Agenda

**DOI:** 10.3389/fsoc.2021.631537

**Published:** 2021-08-19

**Authors:** Y. N. Arifa, S. El Baroudi, S. N. Khapova

**Affiliations:** Department of Management and Organisation, School of Business and Economics, Vrije Universiteit Amsterdam, Amsterdam, Netherlands

**Keywords:** motivations, global mobilty, self-initiated expatriate, assigned expatriate, expatriation

## Abstract

For two decades, individual motivations to expatriate have received substantial attention in the expatriation literature examining self-initiated and assigned expatriation. Recently, however, this literature has changed direction, demonstrating that prior to forming their actual motivations, individuals undergo a process wherein they actively form those motivations. No review has yet unraveled this motivation process, and this systematic literature review fills this gap. Using the Rubicon Action model that discusses the motivation process of expatriation, this article demonstrates that for self-initiated and assigned expatriation, individuals follow similar processes: expatriation expectations are formed; then, they are evaluated; and finally, preferences are built that result in motivations to expatriate. Findings for each stage are discussed in light of their contributions to the expatriation literature. For major gaps, new research suggestions are offered to advance our understanding of the individual motivation process that expats experience prior to forming their motivations to move abroad.

## Introduction

For many countries, expatriation is of paramount importance, especially because it brings in knowledge and talent from abroad, strengthening the competitive advantages of regions and cities within countries (Ridgway and Robson, 2018), and it may even improve a country’s global economic status (Caligiuri and Bonache, 2016). Many countries therefore adopt national and regional strategies to attract talent, as is the case, for example, in the Gulf State of Qatar, where highly skilled expatriates are attracted from Europe, North America, Australia, Egypt, Jordan and the Philippines. The experience, skills and competencies of these expatriates are expected to benefit the country’s stakeholders (Baruch and Forstenlechner, 2017). Also, China welcomes branch campuses of international universities to attract academics from the United Kingdom, Australia and Germany, thus strengthening the country’s human capital (Cai and Hall, 2016). Recognizing the importance of expatriation to countries, researchers have also paid substantial attention to this topic in academic work. This research conducted over the past two decades has identified two main types of expatriation: self-initiated expatriation (SIE) and assigned expatriation (AE) (Andresen et al., 2014; Pinto and Caldas, 2015; Farcas and Goncalves, 2017). Studies have sought to make better sense of these types of expatriation by studying, in particular, individual motivations to expatriate (Suutari and Brewster, 2001; Hippler, 2009; Lee and Kuzhabekova, 2018; Ridgway and Robson, 2018). Perhaps because individual motivations to expatriate to a specific country reflect the country-level factors that attract talent (Ridgway and Robson, 2018), identifying these factors may thus help countries establish effective strategies to attract more talented individuals. Overall, the research findings in this area demonstrate that individuals that undergo SIE and AE are motivated to move abroad by their desire to explore other job opportunities (e.g., Doherty et al., 2011; Froese, 2012) or have a general interest in enhancing their career (Quantin et al., 2012). They may also be motivated to move abroad to live in a country that is economically and politically better developed than their home country (Harry et al., 2017).

Interestingly, such motivations to expatriate are not always driven by serendipity, as extant work suggests (e.g., Doherty, 2013); thus, individuals may actively pursue a strategy to expatriate (Shortland, 2018). This suggests that prior to actual expatriation, individuals go through a process that influences their motivation to expatriate. Confirming this thinking, Glassock and Fee (2015) demonstrate that individuals use social media to actively look for vacancies abroad, a practice that likely influences their motivation to expatriate to a specific country because social media can provide information about job markets in different countries. Likewise, Harris and Brewster (1999) and Shortland (2018) argue that managers utilize their informal networks to look for opportunities to work abroad, which also likely influences their actual motivation to move and work abroad because they can evaluate whether expatriation will benefit their careers (Pinto and Caldas, 2015). Thus, not only do individuals go through a process that influences how and why specific expatriation motivations are formed, but they also seem to have an active role in guiding this process (i.e., triggering their own motivations to expatriate). While this knowledge is clearly discussed in the extant expatriation literature, no review has yet unraveled this individual motivation process (e.g., Al Ariss and Crowley-Henry, 2013; Doherty, 2013; Andresen et al., 2014; Caligiuri and Bonache, 2016; Farcas and Goncalves, 2017). Therefore, it is not known how and why individuals form specific motivations to expatriate and what their active role is in forming their own motivation. The purpose of this paper is to fill in this gap by systematically reviewing articles examining individuals’ motivations to expatriate *via* SIE and AE. By doing so, we particularly focus on 1) whether the articles discuss the process that individuals go through in forming their motivations to expatriate and 2) whether they have an active role in forming their own motivations. To unravel this motivation process, we draw upon the Rubicon model of action phases (Heckhausen and Gollwitzer 1987; Heckhausen and Heckhausen, 2018), which discusses the entire decision-making process of individual expatriation. According to Andresen et al. (2014), motivations are formed in this model through a series of steps: individuals actively form their expectations towards expatriation, then take an active role in evaluating their options to move as SIEs or AEs, and then actively build their preferences for moving somewhere. According to the authors, both SIEs and AEs go through the same stages, and hence, the motivation process is supposed to be the same for both groups.

This article makes two important contributions to the expatriation literature. First, it contributes to extant literature review papers on expatriation motivations by unraveling the process behind the actual motivations. We therefore provide greater understanding of the process that individuals go through when they are pushed towards expatriation (Doherty, 2013; Glassock and Fee, 2015). Second, we aim to demonstrate that as individuals go through that process, they have an active role in forming their own motivations to expatriate. We therefore contribute to extant work that argues that motivations to expatriate are driven by more than just serendipity (e.g., Doherty, 2013) by providing explanations for why this is indeed the case. In the remainder of this article, we define SIE and AE and provide a brief overview of their motivations to expatriate, followed by an explanation of the motivation process in expatriation. We then discuss how we have conducted our review and present our findings. Finally, we discuss future research areas that will further enhance our understanding of how and why motivations to expatriate are formed.

### Defining Self-Initiated and Assigned Expatriation

#### Self-Initiated Expatriation

We follow Doherty (2013) and Al Ariss and Crowley-Henry (2013), who define SIE as denoting internationally mobile individuals who have moved—through their own agency or through an organizationally supported expatriation—to another country for an indeterminate duration. SIEs are considered migrants if they decide to stay in the host country permanently (Al Ariss and Özbilgin, 2010). Because motivations to undertake expatriation are also influenced by demographic characteristics (e.g., Selmer and Lauring, 2010; Doherty et al., 2011; Lauring et al., 2014), it is worthwhile to mention the demographic characteristics of both SIEs and AEs. SIEs tend to be represented by slightly younger individuals, who may be unmarried or married and accompanied by their spouses in their expatriation (Suutari and Brewster, 2000). Individuals studied under the banner of SIEs include graduates (e.g., Suutari and Brewster, 2001), academics (e.g., Lee and Kuzhabekova, 2018), doctors (e.g., Humphries, et al., 2015), entrepreneurs (e.g., Selmer et al., 2018) and managers, technicians and other professionals (Ewers and Shockley, 2018).

#### Assigned Expatriation

We follow the definition of AE used in prior work but emphasize that individuals do have some choice in accepting or declining job assignments and that they may also actively initiate their own AE (Harris and Brewster, 1999). Hence, we define AE as denoting employees who undertake a sponsored expatriation because they have been assigned to a foreign subsidiary by their parent organization, which was either a result of their own initiative (e.g., Harris and Brewster, 1999) or their employer’s initiative but where they had the choice to accept or decline the offer (Andresen et al., 2014; Cerdin and Selmer, 2014). AEs tend to be slightly older and represented more by married males who are also accompanied by their spouses and families (Suutari and Brewster, 2000). Furthermore, AEs tend to be more represented by top managers (Pinto et al., 2012; Pinto and Caldas, 2015).

### Motivations to Expatriate for SIE and AE

Prior work demonstrates that the motivations of SIEs to move abroad have been largely explained by push and pull factors (e.g., Thorn, 2009; Doherty, 2013; Lee and Kuzhabekova, 2018). SIEs move abroad to improve their lifestyle and quality of life (Marian, 2010); thus, career opportunities, cultural exposure, and economic and political factors are *push factors* motivating them to move abroad (e.g., Richardson and McKenna, 2002; Thorn et al., 2013), while family considerations tend to operate as *pull factors* towards the home context, demotivating individuals from initiating expatriation (Jackson et al., 2005). AEs move abroad to fill managerial and technical positions in the host country (Caligiuri and Bonache, 2016). *Push factors* triggering AEs to expatriate are—in addition to pressure from superiors (Pinto et al., 2012; Pinto and Caldas, 2015)—gaining new challenging international work experience, progressing in one’s career and wanting to learn more about oneself (Hippler, 2009). Similar to SIEs, AEs are concerned with pursuing personal and professional development (Baruch and Forstenlechner, 2017). AEs are also concerned with their partner’s and children’s attitude towards relocation; hence, family considerations tend to also operate as *pull factors* towards the home context, triggering individuals to decline international job offers (Doherty et al., 2011; Froese, 2012). Indeed, Doherty and her collaborators (2011) compared the motivations of SIEs and AEs and concluded that career considerations may be important for both groups but are significantly more important to AEs because their international experience is coupled with career development and progression. For SIEs, the status of the host country is a much stronger *pull factor* towards expatriation, likely influencing where individuals decide to pursue their career path. Because career motivations still apply to both SIEs and AEs and because cultural, economic and political factors are country-level factors attracting expats (Jackson et al., 2005; Doherty et al., 2011), we use the categories *career, economy*, *politics* and *culture* to analyze and report our findings regarding the motivations of SIEs and AEs to go abroad and the processes underlying these motivations. Our career category involves motivations related to the subjective career, which is an individual’s sense or evaluation of his or her own career needs and development (Volmer and Spurk, 2011); this concept refers, for example, to career aspirations, employment security and access to learning and development (Arthur et al., 2005). This category also involves motivations related to one’s objective career, which refers to visible indicators of individuals’ career positions, situations and status (Arthur et al., 2005); it involves income, family situation, task attributes, mobility and job level (van Maanen, 1977, p. 9). Our economy category includes motivations derived from a country’s wealth level, which refers, for example, to regulations that exempt residents from paying taxes (Humphries et al., 2015), leaving them with a higher net income (Alonso-Garbayo and Maben, 2009). Our politics category involves motivations derived from factors relating to the politics of a country (Thorn, 2009), such as immigration policies and the freedom to practice different religions (Alonso-Garbayo and Maben, 2009), such as its food, language, habits and any other cultural activities.

### Motivation Process

Before actual expatriation motivations are formed, both SIEs and AEs go through a similar process where expatriation expectations are determined, alternatives are evaluated and preferences are built (Andresen et al., 2014). This motivation process has been explained in extant work using the Rubicon model of action phases (Heckhausen and Gollwitzer, 1987; Andresen et al., 2014; Heckhausen and Heckhausen, 2018; Andresen et al., 2014), where in the first stage of the process, individuals start creating a diffuse idea about the benefits of moving abroad in order to address their individual motivations; this idea forms their *expatriation expectations*. Individuals have an active role triggering their own expectations, as individuals’ perceptions about the benefits of moving abroad develop when they are making sense of the expatriation experiences of others (Kim, 2010). SIEs tend to form expectations by using input from multiple external sources, such as friends, family and the internet (Glassock and Fee, 2015). On the other hand, in forming their expatriation expectations, AEs derive input and clues from colleagues who have already been sent on similar international postings (Pinto and Caldas, 2015). Once *expectations* are formed, individuals continue in the second stage by *evaluating* their options to expatriate (Andresen et al., 2014). This stage implies that they can consider taking the initiative to apply for a job abroad without seeking any organizational support or assistance (SIE) (Suutari and Brewster, 2000). If individuals perceive that such actions will likely be unsuccessful, they may consider seeking assistance from recruitment agencies (Farcas and Goncalves, 2017), use social networks (i.e., friends, family) to find a job abroad (Baruch and Forstenlechner, 2017), or seek support from a company that is willing to support their expatriation (Alonso-Garbayo and Maben, 2009). This company could be the individual’s current employer (AE) (Cuhlova, 2017) or a company abroad seeking to recruit foreigners (SIE) (e.g., Alonso-Garbayo and Maben, 2009). Thus, in this stage, tools and other resources are likely used to evaluate options for expatriation.

The final stage, where *preferences* are built, is influenced by valence and expectancy parameters (Vroom, 1964). Valence can be interpreted as the anticipated satisfaction with an outcome, whereas expectancy can be interpreted as an action or effort leading to the preferred outcome (Vroom, 1964). In terms of expatriation, this definition implies that individuals prefer to expatriate to a country where they expect to have the most opportunities to address their individual and family needs. Such a country will be a preferred location if it offers more job opportunities, higher salaries and better assignment packages than other countries, as well as a safe environment to raise a family (Jackson et al., 2005; Froese, 2012; Lee and Kuzhabekova, 2018; Richardson and Wong, 2018).

### Methodology

The purpose of a systematic literature review is to develop conceptual consolidation across a fragmented field of study and to remove subjectivity by using a predefined selection algorithm (Tranfield et al., 2003). In this sense, we also systematically reviewed articles about individual motivations to go abroad to unravel the individual process behind the actual motivations. In line with Tranfield et al. (2003) systematic review methodology, we also performed the three steps of data collection, data analysis and reporting the findings, which we discuss further below.

#### Data Collection

**FIGURE 1:**
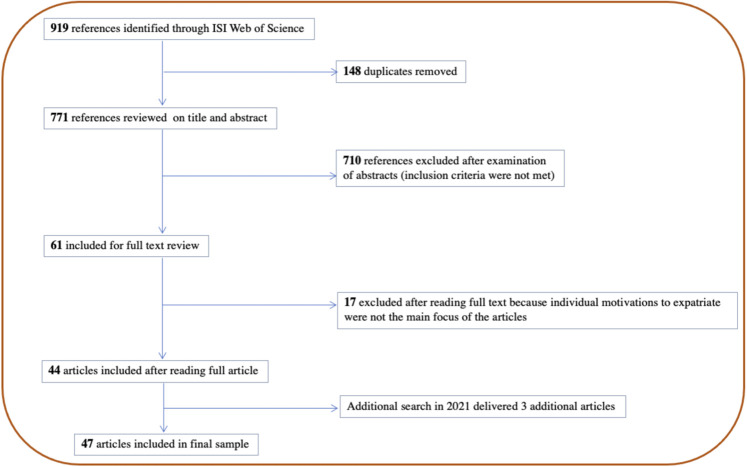
Data collection.

We conducted a series of searches using the ISI Web of Knowledge Database. This database has been used in prior similar work (e.g., Al-Ariss and Crowley-Henry, 2013; Farcas and Goncalves, 2017; Davda et al., 2018) because it includes all journals that have an impact factor and are listed in the Social Science Citation Index; hence, these journals are recognized to have an impact in the expatriation field. We found that the first article examining motivations to expatriate was published in 1994. Our initial search started in 2019 and was therefore focused on identifying articles that were published in the period between 1994 and 2019. We limited the scope of our review by using the key words “expatriate”, “migrant” and “digital nomad” combined with the additional key words “motive”, “motiv”, and “reason” because these terms are mainly used in articles studying motivations in the expatriation literature (e.g., Doherty et al., 2011; Harry et al., 2017). This search returned 919 results. Then, 148 duplicate articles were removed automatically by the web of science, leaving us with 771 articles. We then proceeded to read the abstracts of all 771 articles and excluded another 710 articles because the topics of those articles did not match the purpose of our review. The excluded articles discussed topics such as medical tourism behavior (Mathijsen, 2019), psychological contract perspectives on expatriation failure (Kumarika Perera et al., 2017) and cultural leadership behavior (Kennedy, 2018). All remaining 61 articles were included for full text review, but only 44 articles were included in the final sample because these articles examine individual motivations to expatriate, are empirical studies (i.e., quantitative and qualitative studies) and are written in English. Therefore, literature review articles and empirical studies written in a language other than English were excluded from the final sample. In 2021, we followed the same search strategy to explore whether new articles were published between 2019 and 2021. This search added two articles to the final sample. One additional article was added as it was suggested by a reviewer. The final sample includes 47 articles. Figure 1 shows our data collection visually, and Table 1 includes a bibliography of all included articles.

**TABLE 1:** Overview of articles included in final sample.

No	Article	Author	Journal
1	Self-initiated academics expatriates: inherent demographics and reasons to expatriate	Selmer and Lauring (2010)	European management review
2	Reverse flow in academics mobility from core to periphery: motivations of international faculty working in Kazakhstan	Lee and Kuzhabekova (2018)	Higher education
3	Expatriate academics in Malaysia: motivation, adjustment and retention	Richardson and Wong (2018)	Journal of management development
4	The decision-making processes of self-initiated expatriates: a consumer behavior approach	Glassock and Fee (2015)	Journal of global mobility
5	Motivations, expectations and experiences of expatriate academic staff on an international branch campus in China	Cai and Hall (2016)	Journal of studies in international education
6	Motivation and adjustment of self-initiated expatriates: The case of expatriate academics in South Korea	Froese (2012)	The international journal of human resources management
7	Business or pleasure? Blurring relocation categories and motivation patterns among expatriates	Lauring et al. (2014)	Scandinavian journal of hospitality and tourism
8	Reasons to expatriate and work outcomes of self-initated expatriates	Selmer and Lauring (2012)	Personnel review
9	Acquired demographics and reasons to relocate among self- initiated expatriates	Selmer and Lauring (2011)	The international journal of human resources management
10	Global competition for scientific talent: evidence from location decisions of PhDs and postdocs in 16 countries	Stephan et al. (2016)	Industrial and corporate change
11	Internationally recruited nurses from India and the Philippines in the United Kingdom: the decision to emigrate	Alonso-Garbayo and Maben (2009)	Human resources for health
12	Applying a typology of health worker migration to non-EU migrant doctors in Ireland	Humphries et al. (2015)	Human resources for health
13	Comparison of British and French expatriate doctors characteristics and motivations	Quantin et al. (2012)	BMJ open
14	The importance of intelligent career and location considerations	Dickmann and Mills (2010)	Personnel review
15	Experiences of women professionals abroad: Comparisons across Japan, China and Turkey	Napier and Taylor (2002)	International journal of human resource
16	Compelled to go abroad? Motives and outcomes of international assignments	Pinto et al. (2012)	The international journal of human resource management
17	Making sense of expatriation	Pinto and Caldas (2015)	Management research
18	Motivations and cross-cultural adaptation of self-initiated expatriates, assigned expatriates and immigrant workers: the case of Portuegese migrant workers in the United Kingdsom	Farcas and Goncalves (2017)	Journal of cross-cultural psychology
19	Exploring the Dynamics of New Zealand’s Talent Flow	Jackson et al. (2005)	New Zealand journal of psychology
20	Managerial willingness to assume traveling, short-term and long-term global assignments	Konopaske et al. (2009)	Management international review
21	The coffee-machine system: how international selection really works	Harris and Brewster (1999)	International journal of human resource management
22	Exploring the motives of company-backed and self-initiated expatriates	Doherty et al. (2011)	International journal of human resource management
23	Motivational factors of Czech corporate expatriates to an international assignment	Cuhlova (2017)	Ad Alta: Journal of interdisciplinary research
24	Female expatriates’ motivations and challenges: the case of oil and gas	Shortland (2018)	Gender in management: An international journal
25	Analysis of expatriation process in a Slovenian company	Martins and Bernik (2017)	Organizacija
26	Global careers in the Arabian Gulf	Baruch and Forstenlechner (2017)	Career development international
27	Trading places: French highly skilled migrants negotiating mobility and emplacement in London	Ryan and Mulholland (2014)	Journal of ethnic and migration studies
28	Women’s self-initiated expatriation as a career option and its ethical issues	Tharenou (2010)	Journal of business ethics
29	Why do they go? Empirical evidence of employees’ motives for seeking or accepting relocation	Hippler (2009)	The international journal of human resource management
30	Digital nomads-a quest for holistic freedom in work and leisure	Reichenberger (2018)	Annals of leisure research
31	The relative importance of motives for international self-initiated mobility	Thorn (2009)	Career development international
32	Using narratives to understand the motivational factors and experience of being a self-initiated academic expatriate in South Africa	Harry et al. (2017)	SA journal of human resource management
33	Overseas nurses’ motivations for working in the United Kingdom: globalization and life politics	Larsen et al. (2005)	Work, employment and society
34	Attracting and retatining expatriates in Qatar during an era of uncertainty: Would you stay or would you go?	Ewers and Shockley (2018)	Population, space and place
35	A passage to France: skilled Indian SIEs in transition	Mielly et al. (2017)	Critical perspectives in international business
36	Motivational diversity and the sense of ill-treatment back home among the United Kingdom’s migrant workers from Turkey: a cross-intersectional approach	Cam (2017)	Economic and industrial democracy
37	Motivation for migration and economic success	Winter-Ebmer (1994)	Journal of economic psychology
38	Social networks of Portuguese self-initiated expatriates	Pinto and Araujo (2016)	Journal of management development
39	Exploring the motivation and willingness of self-initiated expatriates in the civil engineering industry, when considering employment in Qatar	Ridgway and Robson (2018)	Human resource development international
40	Qualified immigrants’ success: Exploring the motivation to migrate and to integrate	Cerdin et al. (2014)	Journal of international business studies
41	Migration reasons, traits and entrepreneurial motivation of African immigrant entrepreneurs	Khosa and Kalitanyi (2015)	Journal of enterprising communities: people and places in the global economy
42	Why do they go? Empirical evidence of employees’ motives for seeking or accepting relocation	Hippler (2009)	The international journal of human resource management
43	Consumption of young xin yimin: The role of identity and tacit motives	Ahmed et al. (2015)	Australasian marketing journal
44	Going abroad to play: Motivations, challenges, and support of sports expatriates	van Bakel and Salzbrenner (2019)	Thunderbird international business review
45	Nursing emigration in the United Kingdom: A qualitative exploration of the Spanish nursing community	Rodriguez-Arrastia et al. (2021)	Nursing open
46	The freedom trap: digital nomads and the use of disciplining practices to manage work/leisure boundaries	Cook. (2020)	Information technology and tourism
47	Expectancies and motivational goals of self-initiated expatriates as predictors of subjective assignment achievements and success	Pinto et al. (2020)	Management research review

#### Data Analysis

To analyze our data, we set up a table with thematic codes that included the 1) Name (s) of the author (s); 2) Title; 3) Year of publication; 4) Journal Title; 5) Methodology; 6) Sample; 7) Expatriation type (SIE or AE) 8) Gender; 9) Occupation; (10) Motivation to expatriate; 11) Push and pull factors influencing the motivation process of all four motivations; and 12) Country, (i.e., home or host country). The motivation categories were derived from prior work examining motivations to expatriate and included, as discussed above, career, economy, politics and culture. Finally, the tables were filled in for the self-initiated and assigned expatriates separately.

### Expatriation Motivations and Processes

#### Findings

##### Career

We found thirty-four articles identifying career as a motivation to expatriate for both SIEs and AEs (SIE, N = 23; AE, N = 11). Among these articles, thirty-four discuss expatriation expectations, twenty-nine discuss how individuals expatriate (as SIE or AE), and fifteen demonstrate that individuals developed preferences to expatriate to a specific country. Below, we discuss our findings by applying them to the three stages of the motivation process.

*Forming expectations.* Focusing on one specific group of employees, ten articles demonstrate that academics expect that expatriation could offer more opportunities to develop their academic careers. For instance, studies by Lauring et al. (2014) and Selmer and Lauring (2011, 2012) highlight that academics expect career enhancement during their expatriation. Furthermore, Selmer and Lauring (2010) demonstrate that there is an association between age and career development, which implies—according to the authors—that younger academics expect to find more career development opportunities once they expatriate. Studies by Stephan, Franzoni and Scellato (2016), Lee and Kuzhabekova (2018) and Richardson and Wong (2018) indicate that academics expect to find more opportunities to engage in research if they expatriate to another country. According to Lee and Kuzhabekova (2018), such expectations are coupled with expectations of finding a better balance between teaching and research at universities abroad. Lee and Kuzhabekova (2018) also emphasize that academics expect to contribute to new institutions by developing new programs while working abroad. Similarly, other studies find that academics intend to become involved in new projects when they move abroad (Glassock and Fee, 2015; Cai and Hall, 2016). Academics from the United States indicate that they expect to find more job opportunities abroad compared to their home country, where a surplus of doctorates creates a highly competitive job market (Froese, 2012; Lee and Kuzhabekova, 2018).

According to four articles, other employees specified as healthcare professionals expected to address several career needs if they moved abroad. For example, Alonso-Garbayo and Maben (2009), Quantin et al. (2012) and Rodriguez-Arrastia et al. (2021) indicate that healthcare professionals expect to find a work environment with higher standards, where they can gain new knowledge and develop new skills. In a similar vein, Humphries et al. (2015) demonstrate that healthcare professionals expect to have opportunities for postgraduate training if they expatriate, which would benefit their careers. In addition, Quantin et al. (2012) clearly demonstrate that healthcare professionals located in different countries have different expectations of expatriation. While French doctors especially expect to find improved work conditions abroad, United Kingdom doctors expect to find interesting new positions.

Another group of employees identified in eleven articles includes managers, directors and vice presidents. In this group, expatriation expectations were found to be linked to career progression expectations (e.g., Napier and Taylor, 2002; Jackson et al., 2005; Konopaske et al., 2009; Dickmann and Mills, 2010; Pinto et al., 2012; Pinto and Caldas, 2015; Farcas and Goncalves, 2017). In this case, career progression expectations refer to expectations of finding new work responsibilities and new professional challenges while working abroad (Dickmann and Mills, 2010; Farcas and Goncalves, 2017). In a similar vein, financial professionals, engineers, designers, lawyers, senior care assistants, and government officials are discussed in four articles, in which it is argued that they expect to meet their career progression expectations if they move abroad (e.g., Doherty et al., 2011; Cuhlova, 2017; Shortland, 2018) or to upgrade their knowledge and improve their general skills (Martins and Bernik, 2017).

Other skilled employees for whom their profession was not specified in the articles also highlight career as a reason to move abroad, as mentioned in eight articles. For instance, they expect to find more career advancement opportunities when they return back home (Baruch and Forstenlechner, 2017; Ryan and Mulholland, 2014, Tharenou, 2010; Hippler, 2009, Thorn, 2009). Another group of employees—athletes—are identified in one article, which discusses that this group expects to advance in their careers by playing professionally if they move abroad (van Bakel and Salzbrenner, 2019). We found two articles studying digital nomads. This group’s expatriation expectations are linked with their career expectations because they expect to find more flexible work arrangements abroad (Reichenberger, 2018; Cook, 2020). Finally, in another recent article Pinto et al. (2020) show that SIEs (different professions) expected to pursuit performance goals, but this was more likely the case for SIEs who had greater confidence in their ability to live and work abroad. The above-discussed articles clearly demonstrate that individuals expect their needs to be fulfilled if they move abroad, but none of these articles demonstrate whether individuals have an active role in triggering their own expectations. That is, it is not known how individuals form their expectations.

*Evaluating options*. Among the ten articles studying academics’ motivations to expatriate for career reasons, only Richardson and Wong (2018) explain that some academics have moved to Malaysia as SIEs because they received job offers from a university. The authors do not discuss whether these academics also considered expatriating as AEs. Similarly, Cai and Hall (2016) discuss how some academics moved to China as AEs because they were required to fulfill positions in the international branch campus; they accepted the offer because the international branch campus offers higher salaries than local Chinese universities. Glassock and Fee (2015) provide more insights into how academics use multiple external sources to evaluate how they should expatriate for career reasons. For instance, their study reveals that academics use the internet to search for job vacancies abroad and ask for advice from family and friends. Alumnae networks are also used to find expatriates and gather tips and tricks about how to expatriate. Academics also seek jobs abroad through recruitment agencies and by attending open days organized by recruitment agencies. Eventually, this search process triggers them to expatriate as SIEs, as illustrated in the article. Of the four articles studying healthcare professionals, only Alonso-Garbayo and Maben (2009) discuss how nurses took the initiative to actively look for job opportunities in specific countries using recruitment agencies in their home country. However, it is not discussed whether they evaluated other options to expatriate.

By contrast, among the eight articles studying managers, directors and vice-presidents, five articles discuss in greater depth how this group of employees evaluates options to expatriate as an SIE or AE for career reasons. A study by Farcas and Goncalves (2017) reveals that managers located in Portugal searched for jobs in the United Kingdom using social media (i.e., LinkedIn) and other tools such as VidaEdu and INOV Contacto, which are programs offering educational and work opportunities abroad for highly educated Portuguese citizens who are interested in gaining international work experience. These managers also contacted recruitment agencies and used personal contacts who were already working in the United Kingdom to help them find a job. Farcas and Goncalves (2017) demonstrate that managers decided to expatriate as SIEs because of the deteriorating professional situation in their home country, and they took the initiative to look for jobs abroad themselves. Pinto and Caldas (2015) demonstrate that individuals expatriate as AEs because they were assigned to move abroad. Harris and Brewster (1999) found that managers nominate themselves to be sent abroad and use informal networks and conversations with influential individuals to initiate their AE. Only the article by Pinto et al. (2012) reveals that expatriation was initiated by the company and required commitment from the assignee, which indicates that in this particular case, different options to expatriate were not evaluated prior to expatriation. Four articles studying financial professionals, engineers, designers, lawyers, senior care assistants, and government officials demonstrate that individuals expatriate as AEs for career reasons; however, these articles do not discuss whether individuals evaluate options to expatriate as SIEs (e.g., Cuhlova, 2017; Martins and Bernik, 2017; Shortland, 2018). Only one article of the four (e.g., Doherty et al., 2011) indicates that individuals moved as SIEs. Shortland (2018) reveals that once individuals decide to expatriate as AEs, the selection process relies heavily on personal contacts, which implies that individuals should take an active role in asking for support from their professional contacts to increase the likelihood of being selected. Other skilled employees for whom their profession was not specified are discussed in four articles, but only an article by Baruch and Forstenlechner (2017) reveals that SIEs arrive in the host country through their professional networks. Their study reveals that individuals from North African, European and American countries hear about potential job vacancies abroad from their friends and other personal contacts, while individuals from Southeastern Asian countries receive information solely from family members, and individuals from other African countries use the internet to find information about jobs abroad. Finally, the article on athletes demonstrates that this group of professionals worked with a recruitment agency to arrange a contract abroad and to secure assistance while dealing with practical issues (van Bakel and Salzbrenner, 2019).

*Building preferences.* Referring back to Selmer and Lauring (2010), who focus on the career expectations of younger academics, the authors also demonstrate that salary and other financial incentives play an important role in influencing the actual expatriation of these individuals, which implies that their preferences to move abroad are still developed based on subjective and objective career needs (Biemann and Braakmann, 2013). Lee and Kuzhabekova (2018) reveal that marital status, age, work conditions and the job market are factors influencing how expatriates developed their preference to work overseas. In terms of accepting international assignments, the authors argue that single and young academics are more likely to actively develop a preference to move abroad because they are more adventurous and thus more enthusiastic about international experiences than their older peers. Referring back to Glassock and Fee (2015), the authors also identify multiple criteria that academics use when selecting a country for expatriation. These criteria include whether they can speak and understand the language of the host country, whether the country is safe, whether the economic and political conditions are stable, and whether the standards of living are affordable. The authors also reveal that salary plays an important role in influencing the preference to move and that individuals seem to consider visiting the preferred country to gain real experience prior to determining their final preference. In a similar vein, Cai and Hall (2016) reveal that young academics prefer to move to China because they have previously visited the country or lived there before. Pinto and Caldas (2015) demonstrate that individuals expatriate as AEs because they have been assigned to move abroad. This article also reveals that this particular group preferred to accept their assignments because they had evaluated whether the international experience would add value to their personal and professional lives based on the experiences of their colleagues who had already worked on assignments abroad. Focusing on healthcare professionals, we found only one article by Alonso-Garbayo and Maben (2009) demonstrating that these professionals, specifically nurses, developed preferences for a specific host country based on historical ties between that country and their home. For example, nurses from India preferred to expatriate to the United Kingdom over the United States because their home country was colonized by the United Kingdom in the past, and the migration procedure would therefore be less complicated in the United Kingdom than in the United States. Muslim nurses preferred to move to the United Kingdom because, in addition to career reasons, they would also be able to practice their religion freely there, including at work. Furthermore, preference for a country was also built based on whether nurses had a social network in the country. Studies examining other highly educated and experienced professionals for whom their profession was not specified demonstrate that married women preferred to accept expatriation assignments that were long-term and offered the highest financial compensation, as well as opportunities to maintain a healthy work-family balance (Shortland, 2018). Other individuals prefer to accept an international assignment where they can experience the most personal, professional and salary growth (Cuhlova, 2017). Baruch and Forstenlechner (2017) explore differences between ethnicities and show that Westerners solely prioritize the career opportunities of expatriation, whereas individuals from Muslim countries prefer moving to Muslim countries that are not influenced by religious extremism to raise their families. SIEs tend to also compare locations and form preferences for the location that has the best reputation. SIEs clearly argue that expatriating to the UAE offers them more benefits compared to Saudi Arabia and Russia in terms of the country’s reputation. Finally, Thorn (2009) demonstrates that while men formed preferences to move somewhere where they could address their career needs, other factors such as the economy, quality of life and the political environment tend to influence their actual preferences for a host destination.

##### Economy

Our review reveals that compared to career motivations, far fewer articles focus on whether individuals are motivated to expatriate for economic reasons. Eleven articles examined this topic, of which all eleven discuss expatriation expectations and how individuals evaluate options to move abroad and seven demonstrate that individuals use economic criteria when forming their preferences to expatriate to a specific country.

*Forming expectations.* Studying different types of employees, eleven articles demonstrate that individuals are driven to expatriate to a country that is economically well developed to improve their own financial conditions. For instance, Harry et al. (2017) reveal that academics from poor countries expect to receive higher salaries and remuneration in South Africa because the country is better economically developed than their home countries. Likewise, academics from non-EU countries are also interested in financial incentives offered by EU countries (Selmer and Lauring, 2011). In another study, healthcare professionals expect to find economic advantages in Ireland, which is expected to offer tax exemptions and higher salaries (Alonso-Garbayo and Maben, 2009; Humphries et al., 2015). In a similar vein, Larsen et al. (2005) note that nurses expect to be able to send more remittances back home if they expatriate to the United Kingdom. Two articles show that Qatar and London are perceived to be economically well developed by managers (e.g., Dickmann and Mills, 2010; Ewers and Shockley, 2018), which is likely why they expect higher salaries when moving. Mielly et al. (2017) reveal that Indian employees expect France to be five times better economically developed than India. According to these Indian employees, France could offer them and their families a more prestigious life. Similarly, other skilled employees from New Zealand expect Asian countries to offer them economic advantages (Thorn, 2009), and employees from Turkey expect the United Kingdom to provide them with economic benefits because it offers comparatively more business opportunities (Cam, 2017). Employees from Yugoslavia and Turkey expect to earn more income by moving to Austria (Winter-Ebmer, 1994), likely also because it is wealthier than their home country. Although the eleven articles clearly demonstrate that individuals expect to benefit from the economic conditions in their host countries, none of the articles demonstrate how these individuals formed their expectations, which was also identified as a gap in the career motivation category.

*Evaluating options.* Eleven articles indicate that academics, healthcare professionals, managers, directors and other skilled employees expatriated as SIEs for economic reasons (e.g., Larsen et al., 2005; Alonso-Garbayo and Maben, 2009; Thorn, 2009; Dickmann and Mills, 2010; Selmer and Lauring, 2011; Ryan and Mulholland, 2014; Humphries et al., 2015; Harry et al., 2017; Mielly et al., 2017; Ewers and Shockley, 2018), and one article indicates that managers also expatriated as AEs (Dickmann and Mills, 2010). However, none of these articles discuss whether the individuals evaluated expatriation options. Alonso-Garbayo and Maben (2009) revealed that nurses used recruitment agencies in their home countries to obtain jobs abroad. Cam (2017) provides some insights into the means used by SIEs to evaluate their expatriation options by discussing how individuals use their personal social network in the host country (United Kingdom) to find assistance during their expatriation.

*Building preferences*. Seven articles demonstrate that individuals formed preferences to expatriate to a country that is economically better developed compared to their home country (e.g., Winter-Ebmer, 1994; Larsen et al., 2005; Alonso-Garbayo and Maben, 2009; Dickmann and Mills, 2010; Ryan and Mulholland, 2014; Mielly et al., 2017; Ewers and Shockley, 2018) and that offers additional benefits such as new cultural exposure (Larsen et al., 2005), local security (Ewers and Shockley, 2018), favorable opportunities to save money (Alonso-Garbayo and Maben, 2009) and a clear and relatively easy immigration procedure (Ryan and Mulholland, 2014; Mielly et al., 2017). Only one article by Alonso-Garbayo and Maben (2009) discusses SIEs’ active role in forming preferences to move to a specific location; the authors argue that SIEs took the initiative to seek information about countries from the companies for which they aimed to work in the host country.

##### Politics

We found ten articles discussing politics as a motivation to expatriate, but solely for SIEs (N = 10). Among these articles, nine discuss expatriation expectations, ten indicate that individuals evaluate options to move abroad, and five discuss how individuals form their preferences to move to a specific country. We further discuss our findings below.

*Forming expectations.* Two articles reveal that academics expect to find a safer political environment by moving to another country. For example, Richardson and Wong (2018) demonstrate that Iraqi and Iranian academics expect to find a more stable political environment in Malaysia. Similarly, in another article, Harry et al. (2017) reveal that academics from poor countries move abroad not only for economic reasons but also because they expect to find more political stability. Additionally, healthcare professionals appear to have expectations of finding political stability abroad, as demonstrated in another two articles (e.g., Alonso-Garbayo and Maben, 2009; Pinto and Araujo, 2016). Specifically, Pinto and Araujo (2016) indicate that healthcare professionals from Portugal expect to find a better political situation abroad. The study of Alonso-Garbayo and Maben (2009) demonstrates that nurses from India expect to be able to practice their religion freely if they expatriate to the United Kingdom. In another three articles, engineers are found to have similar expectations regarding finding a safer political environment abroad and expect to benefit from the political system in the host country. For instance, Ridgway and Robson (2018) mention that Syrian SIEs want to remain overseas due to political conflicts in their home country and they expect to avoid obligatory participation in military service back home. Mielly et al. (2017) demonstrate that Indian engineers expect to be able to apply for the French nationality when they expatriate to France. Cerdin et al. (2014) reveal that female engineers expect to gain more freedom and rights living in a democratic country. Entrepreneurs from Zimbabwe and Nigeria who moved to South Africa were found to have similar expectations about finding political stability and benefits abroad, as discussed in Khosa and Kalitanyi (2015) article. Finally, Thorn (2009) demonstrates that highly educated individuals for whom their profession was not specified expect to benefit from immigration policies; for example, they expect to receive a working visa easily in certain countries. Although all nine articles clearly discuss expatriation expectations of finding political safety and political advantages abroad, none reveal how individuals have formed these expectations, which is a gap also identified in the prior two expatriation motivations *career* and *economy*.

*Evaluating options.* All ten articles noted that academics, healthcare professionals, engineers, entrepreneurs and highly skilled migrants moved as SIEs (Alonso-Garbayo and Maben, 2009; Thorn, 2009; Cerdin et al., 2014; Khosa and Kalitanyi, 2015; Pinto and Araujo, 2016; Cam, 2017; Harry et al., 2017; Mielly et al., 2017; Richardson and Wong, 2018; Ridgway and Robson, 2018). However, among those articles, only two discuss how individuals evaluated their options for moving as SIEs, and none discuss whether options for moving as AEs were evaluated.

*Building preferences.* We found five articles discussing how SIEs formed their preferences to move to a specific country; they did this by comparing political situations between home and host countries (Pinto and Araujo, 2016; Harry et al., 2017; Mielly et al., 2017) and comparing which country would offer the most freedom to practice other religions (Alonso-Garbayo and Maben, 2009; Cam, 2017).

##### Culture

Our review reveals that ten articles demonstrate that individuals are motivated to expatriate for cultural reasons. Among these articles, expatriation expectations are discussed in eight, ten articles indicate whether individuals moved as SIEs or AEs, and five articles discuss how individuals formed their preferences to move to a specific country. The findings are elaborated in more detail below.

*Forming expectations.* Eight articles reveal that individuals expect to be exposed to a different culture during their stay abroad, and they expect this exposure to enrich their personal lives. Academics, managers and engineers are attracted to different types of food, art and languages (Cerdin et al., 2014; Glassock and Fee, 2015; Cai and Hall, 2016; Mielly et al., 2017). In another article, Ahmed et al. (2015) demonstrate that Chinese employees holding different positions in different fields and industries expect that expatriation to a Western country will help develop their self-confidence and social identity, particularly because the Western culture is associated with prestige and honor. In another article, Hippler (2009) demonstrates that assigned expatriates expect to broaden their horizons through new experiences, especially through living in another culture in another country, region or city. Ryan and Mulholland (2014) reveal that Polish employees moved to London because they expected to learn English. Digital nomads also move abroad because they expect to live with people from different cultures, as discussed in Reichenberger’s article (2018). As with the previously discussed expatriation motivations, we also identified a similar gap in the sense that none of the articles discuss how individuals formed their expatriation expectations.

*Evaluating options.* Among ten articles, seven indicate that academics, healthcare professionals, managers, engineers, other skilled employees and digital nomads moved as SIEs (Alonso-Garbayo and Maben, 2009; Cerdin et al., 2014; Ryan and Mulholland, 2014; Glassock and Fee, 2015; Mielly et al., 2017; Baruch and Forstenlechner, 2017; Reichenberger, 2018). Another two articles demonstrate that academics and other skilled employees moved as AEs (e.g., Hippler, 2009; Cai and Hall, 2016). We find again that academics and nurses moved as SIEs, as they had searched for vacancies abroad (Alonso-Garbayo and Maben, 2009; Glassock and Fee, 2015). Other skilled professionals decided to move as SIEs because they received assistance from their personal networks in the host country (Baruch and Forstenlechner, 2017). For example, individuals who originated from North Africa and North America used information from friends; those from Southeast Asian countries gained information from family members, and South African SIEs utilized the internet (Baruch and Forstenlechner, 2017). According to Ryan and Mulholland (2014), SIEs also contacted recruitment agencies to find jobs abroad.

*Building preferences.* Five articles demonstrate that individuals formed their preference to move to a new country for cultural reasons. Referring back to Mielly et al. (2017), the authors demonstrate that engineers formed a preference for a location where they could learn a new language and also benefit from rather simple visa application procedures, which they believed to be the case in the United Kingdom. Other skilled employees studied by Baruch and Forstenlechner (2017) preferred to move to the UAE because the country’s population includes a wide range of national and cultural backgrounds, which, according to them, facilitates their spouses’ cultural adaptation. In another article, Ryan and Mulholland (2014) reveal that other skilled employees preferred to move to an English-speaking country and decided to move to London because it offers the greatest variety of cultures. Referring again to Cai and Hall (2016), their article demonstrates that individuals preferred to move to China not only for exposure to a new culture but also to access additional benefits, such as opportunities to pursue their personal and professional ambitions. Finally, Alonso-Garbayo and Maben (2009) demonstrate that nurses formed their preferences to move abroad for cultural reasons and sought locations where they could benefit from other economic and professional advantages. While it is clear that preferences are built, none of these articles demonstrate how individuals actively seek information to build such preferences; this was also identified as a gap in the context of economic motivation, discussed above. A summary of the findings can be found in Tables 2, 3.

**TABLE 2:** Overview of the motivation process for all four motivations and the different types of employees.

Motivation	Type of employees	Forming expectations	Evaluating options	Building preferences
Career	Academics	• Career development (i.e., younger academics)	• SIE	• For locations offering the most opportunities to fulfill subjective and objective career needs
		• More research opportunities and opportunity to build new institutions and programs	• AE	• For locations offering good work conditions and loose job markets (i.e., young and single SIEs)
		• Involvement in new projects	—	• For countries offering international involvement (i.e., moved from US to Kazakhstan)
		• More job opportunities	—	• For countries with a familiar language and, a safe environment (i.e., economic and political stability
		• Opportunity to enhance one’s career and receive promotions	—	• For countries where the individual has lived or travelled before and with opportunities for professional development (i.e., China, young academics)
		—	—	• For countries where institutions offer the most research excellence (i.e., US)
	Healthcare Professionals	• Find work where knowledge can be gained and new skills can be developed	• SIE	• For countries that have historical ties with the home country (i.e., colonization, nurses prefer United Kingdom over United States)
		• Obtain postgraduate training	—	• For countries where religion can be practiced freely (i.e., for Muslim nurses)
		• Improvement in work conditions	—	• For countries where individuals have a social network
		• Interesting job positions (i.e., United Kingdom doctors)	—	—
	Managers, Directors, Vice Presidents	• Career progression	• SIE	• Criteria are whether international experience adds value to personal and professional life
		—	• AE	• Criteria to accept long term assignments are: sense of adventurousness, spouse’s willingness to relocate and financial rewards and benefits
		—	—	• Countries that offer safety and easy adaption to local culture
	Financial Professionals, Engineers, Designers, Lawyers, Senior Care Assistants, Government Officials	• Career progression	• SIE	• Long-term assignment with high salary and work life balance (criteria for married women)
		• Upgrading knowledge and developing working skills	• AE	• Where the highest personal, professional and financial growth can be achieved
	Other Skilled Employees (Professionals)	• Career development	• SIE	• Solely career opportunities matter (criteria for Western expats)
		• Achievement of performance goals	• AE	• For countries without religious extremism, preference for UAE over Saudi Arabia and Russia (criteria for Muslim expats)
		—	—	• For countries with a strong business reputation, where there are more skilled professionals and more networking opportunities
		—	—	• Countries with a stable economy and safer political environment (criteria for men)
	Digital nomads	• Flexible work arrangement	• SIE	• Not discussed
	Sports Professionals (i.e., Athlete)	• Career advancement	• SIE	• Countries offering the most professional leagues such as Spain, Germany, France and Switzerland
Total *N* = 34 articles	—	Total *N* = 34 articles	Total *N* = 29 articles	Total *N* = 15 articles
Economy	Academics	• Higher salaries and remuneration in South Africa, because it is economically better developed compared to the home country	• SIE	• Not discussed
		• Financial incentives offered by the host country	—	—
	Healthcare Professionals	• The host country is able to offer tax exemption and higher salaries	• SIE	• For countries with a strong economy and where a new culture can be experienced (i.e., nurses)
		• Ability to send more remittances back home as the host country (United Kingdom) is economically developed	—	• For countries with better economic conditions
	Managers, Directors	• Host country offers higher total income	• SIE	• Moved to country with a stronger economy and local security as an additional benefit
		—	• AE	—
	Other Skilled Employees	• A more prestigious life in France, because it is five times economically more developed than India	• SIE	• Moved to country with a stronger economy and simple and clear immigration procedure as an additional benefit (criteria for Non-EU nationals)
		• More economic advantage in Asia (i.e., New Zealanders who moved to Asia)	—	• To cities offering low income taxes and low costs of living
		• United Kingdom offers more business opportunities compared to Turkey	—	—
		• Austria offers more income compared to Yugoslavia and Turkey	—	—
Total *N* = 11 articles	—	Total *N* = 11 articles	Total *N* = 11 articles	Total *N* = 7 articles
Politics	Academics	• Finding a safer political environment (i.e., Iraqi and Syrian academics who moved to Malaysia; other academics from poor countries)	• SIE	• Comparing political situations between host and home country
	Healthcare Professionals	• Finding political stability (i.e., Doctors who moved from Africa to Ireland; nurses who moved to United Kingdom)	• SIE	• Comparing political situations between host and home country
		—	—	• Moved to United Kingdom, where nurses can more freely practice their religion
	Engineers	• Benefit from host country’s political system (i.e., avoid obligatory military participation in Syria)	• SIE	• Comparing political situations between host and home country
		• Gaining host country nationality (i.e., Indians who moved to France)	—	—
		• More freedom and rights in democratic country (expectations of women)	—	—
	Entrepreneurs	• Finding political stability in South Africa	• SIE	• Not discussed
	Other Employees (including Highly Educated Expatriates)	• Being able to easily obtain working visa	• SIE	• Not discussed
Total *N* = 10 articles	—	Total *N* = 9 articles	Total *N* = 10 articles	Total *N* = 5 articles
Culture	Academics	• Enrich personal life by being exposed to a different culture (i.e., attracted to food, art and language)	• SIE	• For China because it offers the most opportunities for exposure to a new culture (i.e., Western academics)
	Healthcare Professionals	• Not discussed	• SIE	• Additional cultural reasons
	Managers	• Experience different traditions	• SIE	• Not discussed
	Engineers	• Enrich personal life (i.e., attracted to food, art and language)	• SIE	• To a location where they can learn a new language
	Other Employees (Professionals)	• Broaden horizon by living in a country with a different culture.	• SIE	• For countries with diverse national and cultural backgrounds to facilitate spouses’ cultural adaptation
		• Developing self-confidence and social identity.	—	• To cities offering the most cultural diversity
		• Interested in joining a new city (i.e., Polish employees who moved to London).	• AE	• For countries that are geographically the closest to the home country (i.e., France and London) and English speaking (i.e., London)
		• Experiencing a new culture	—	—
	Digital Nomads	• Being exposed to new cultures (i.e., living in a new environment and to getting to know other people)	• SIE	• Not discussed
Total *N* = 10 articles	—	Total *N* = 8 articles	Total *N* = 9 articles	Total *N* = 5 articles

**TABLE 3:** An overview of the active role of different types of employees during the motivation process for all four motivations.

Motivation	Type of employees	Forming expectations	Evaluating options	Building preferences
Career	Academics	• Not discussed	• Applied and received a job offer from a university abroad (SIE)	• Visiting the country to gain real-life experience prior to expatriation
			• Fulfilling position in international branch campus (AE)	
			• Job search using internet, information from family and friends, alumnae networks and recruitment agencies (SIE)	
	Healthcare Professionals	• Not discussed	• Using recruitment agencies in the home country	• Evaluating whether migration procedure is less complicated in the United Kingdom compared to the US (Indian nurses)
				• Seeking assistance from friends in the host country
	Managers, Directors, Vice Presidents	• Not discussed	• Using social media, friends and recruitment agencies to look for jobs (SIE)	• Making sense through colleagues’ expatriation experience
			• Using informal networks at work to initiate AE	
	Engineers	• Not discussed	• Seeking support from personal contacts at work (AE)	• Not discussed
	Other Employees (Professionals)	• Not discussed	• Looking for a job using professional networks (SIE)	• Not discussed
			• Looking for jobs using friends and personal contacts (SIEs from North Africa, Europe and North America)	
			• Looking for jobs using family (SIEs from Southeast Asian countries)	
			• Looking for jobs using internet (SIEs from African countries)	
	Sports Professionals	• Not discussed	• Worked with agency to get contract abroad and assistance with practical issues	• Not discussed
Total *N* = 33 articles	—	Total *N* = 0 articles	Total *N* = 8 articles	Total *N* = 4 articles
Economy	Academics	• Not discussed	• Not discussed	—
	Healthcare Professionals	• Not discussed	• Using recruitment agencies in the home country	• Evaluating whether migration procedure is less complicated in the United Kingdom compared to the US and seeking information from the company’s representatives in the home country (i.e., Indian nurses)
	Other Employees	• Not discussed	• Using personal social networks in the host country to find jobs as SIEs	• Not discussed
			• Using recruitment agency in the home country	
Total *N* = 11 articles	—	Total *N* = 0 articles	Total *N* = 2 articles	Total *N* = 1 article
Politics	Academics	• Not discussed	• Not discussed	• Not discussed
	Healthcare	• Not discussed	• Not discussed	• Not discussed
	Engineers	• Not discussed	• Not discussed	• Not discussed
	Entrepreneurs	• Not discussed	• Using social networks to find a job and settle in the host country	• Not discussed
	Highly Educated Expatriates	• Not discussed	• Not discussed	• Not discussed
	Migrants	• Not discussed	• Not discussed	• Not discussed
Total *N* = 10 articles	—	Total *N* = 0 articles	Total *N* = 1 article	Total *N* = 0 articles
Culture	Academics	• Not discussed	• Job search using internet, information from family and friends, alumnae networks and recruitment agencies (SIE)	• Not discussed
			• Recruited or appointed to fill opportunities in the host country	
	Healthcare Professionals	• Not discussed	• Not discussed	• Not discussed
	Managers	• Not discussed	• Not discussed	• Not discussed
	Other Employees	• Not discussed	• Looking for a job using professional networks (SIE)	• Not discussed
			• Looking for jobs using friends and personal contacts (SIEs from North Africa, Europe and North America)	
			• Looking for jobs using family (SIEs from Southeast Asian countries)	
			• Looking for jobs using internet (SIEs from African countries)	
			• Contacted recruitment agencies	
Total *N* = 10 articles	—	Total *N* = 0 articles	Total *N* = 4 articles	Total *N* = 0 articles

### Discussion and Future Research Suggestions

The aim of this article was to systematically review literature discussing motivations to expatriate. The specific goals were to 1) unravel the motivation process that individuals go through prior to forming their actual motivations and 2) to identify the active role they have during this process. To achieve these aims, we have discussed the motivation process for the four different expatriation motivations separately in the findings section. In this section, we will connect and discuss the four expatriation motivations together to offer a comprehensive understanding of the existing literature in this area. After discussing each stage of the motivation process, we will discuss the major gaps we found and offer suggestions for addressing them in future research.

#### Forming expectations

As previously discussed, in the Rubicon model of action phases (Heckhausen and Gollwitzer, 1987; Heckhausen and Heckhausen, 2018), expatriation expectations are formed when individuals start creating a diffuse idea about the benefits of moving abroad in order to address their individual needs (Andresen et al., 2014). Our findings reveal that individuals enter this stage when they are dissatisfied with their current situation back home (Harry et al., 2017; Lee and Kuzhabekova, 2018) or when they expect that they can improve their personal situation by expatriating (Selmer and Lauring, 2012; Ewers and Shockley, 2018). Thus, regardless of whether individuals want to move for career, economic, political or cultural reasons, expatriation expectations reflect the factors that push individuals to think about leaving their homes. However, to better make sense of this stage, our review reveals that we cannot treat individuals as a homogenous group and that we must take some important factors into account, such as what kind of employee the individual is as well as his/her home country and gender. We will elaborate further on our argument below.

*Type of employee*. Our review reveals that similar types of employees have common needs that they cannot fulfill in their home countries, which triggers them to form expectations about expatriation. For example, in a number of studies, academics are found to look for research opportunities elsewhere (Stephan et al., 2016; Lee and Kuzhabekova, 2018). In a similar vein, healthcare professionals and managers search for higher salaries (Dickmann and Mills, 2010; Humphries et al., 2015; Ewers and Shockley, 2018). Sports professionals search for new opportunities to advance in their careers (van Bakel and Salzbrenner, 2019). Our review thus reveals that there are common needs shared by individuals who belong to the same group of employees but that common needs may also be shared among individuals belonging to different groups of employees (i.e., healthcare professionals and managers). To understand which individuals enter the first stage of the motivation process, one must thus explore the needs or dissatisfying factors that exist for each type of employee.

*Home country.* Our review also demonstrates that it is worthwhile to consider an individual’s country of residence (prior to expatriation) to make sense of expatriation expectations, as this may facilitate exploring common needs that exist among different types of employees from different countries. For instance, based on their expatriation expectations, it is evident that individuals who come from poorer and less developed countries are more concerned about finding a new home in a new country that is much better developed economically and politically than back home, and as our review demonstrates, this seems to apply to academics, healthcare professionals, engineers and entrepreneurs (Alonso-Garbayo and Maben, 2009; Khosa and Kalitanyi, 2015; Pinto and Araujo, 2016; Harry et al., 2017; Mielly et al., 2017; Richardson and Wong, 2018).

*Gender.* Although we did not find much evidence to argue what role gender plays in this first stage of the motivation process, we do would like to note that females working in male-dominated fields such as engineering may be more triggered to form expectations towards expatriation because they are looking for more opportunities in their industry elsewhere (Cerdin et al., 2014). According to Tharenou (2010), this perception among women may partly come from within, as they should be more confident about their skills and competencies in male-dominated fields. Furthermore, the author argues that if women want to be assigned to expatriate, they must be confident and sell themselves more in the expatriation selection process. Future research is needed to further explore the role that gender plays in the first stage of the motivation process in order to understand what differentiates men from women in how they form expatriation expectations.

*Future research suggestions*. We identified a major gap in the literature, indicating that while individuals form expectations about moving abroad, we do not know how they form such expectations. We recommend that future research addresses this gap by examining how a work environment triggers individuals to form expectations about expatriation. For instance, if individuals work for an employer who regularly assigns employees to international postings, they have more opportunities to make sense of the expatriation experiences of their peers at work (Kim, 2010; Pinto and Caldas, 2015). Thus, if they are looking to address their individual needs, whether these are career related or belong to the other three motivation categories, they likely have more information available with which to form expectations about where and how to fulfill those needs. Another research suggestion is to focus on an individuals’ personal and social network by examining how it triggers individuals to move abroad. Finally, the internet may be another useful tool that individuals can use to form their expatriation expectations (Glassock and Fee, 2015); further research could focus on exploring how social media influences expatriation expectations.

#### Evaluating options

Continuing with the second stage, our review reveals that the literature solely discusses how individuals expatriate (either as SIE or AE) but not whether they engage in a process of evaluating which of the two types of expatriation would offer the most benefits. For instance, our findings reveal that several types of employees, such as academics, managers, directors, vice presidents, financial professionals, engineers, designers, lawyers, senior care assistants and other highly skilled professionals (Hippler, 2009; Dickmann and Mills, 2010; Selmer and Lauring, 2010; Doherty et al., 2011; Cai and Hall, 2016; Baruch and Forstenlechner, 2017; Cuhlova, 2017) expatriate as both SIEs and AEs, and hence, it is likely that they can evaluate both options prior to expatriating. For other types of employees, such as the digital nomad and entrepreneur, our findings reveal that they only expatriate as SIEs, probably because they work individually and are not affiliated with a company that can send them abroad (Khosa and Kalitanyi, 2015; Reichenberger, 2018). To advance our understanding of how individuals go through the second stage of the motivation process, much more research is needed to identify which types of employees have opportunities to travel as both SIEs and AEs to explore whether and how they evaluate the two options prior to expatriation. Our review reveals that existing articles do provide insights into the tools that individuals use to plan for their expatriation when they decide to move as SIEs. Several tools are used by different types of employees who expatriate as SIEs for different reasons (i.e., career, economy, politics and culture). For instance, healthcare professionals, managers, vice presidents, directors and sports professionals seem to look for jobs abroad using recruitment agencies (Alonso-Garbayo and Maben, 2009; Farcas and Goncalves, 2017; van Bakel and Salzbrenner, 2019). Social networks, including family members, alumnae, friends and managers, are used by academics, engineers, and other highly skilled professionals to look for jobs abroad (Baruch and Forstenlechner, 2017; Richardson and Wong, 2018; Shortland, 2018). Finally, the internet is used to apply for jobs individually by academics, managers and other highly skilled professionals (Glassock and Fee, 2015; Baruch and Forstenlechner, 2017; Farcas and Goncalves, 2017).

*Future research suggestions*. We previously recommended that future researchers identify whether and how individuals evaluate their options to expatriate as either SIEs or AEs, and we suggest again that future research may further explore how individuals use their work environment to evaluate options to expatriate as SIEs or AEs. The individual-level factors age and gender should also be taken into account in future research in this second stage of the motivation process, as international assignments are more likely to be given to experienced managers (Pinto et al., 2012). Therefore, younger individuals might be less encouraged to evaluate whether AE is an option to consider, even though they work in a company that sends individuals abroad. Likewise, married females are less encouraged to do so because they are given fewer opportunities to work abroad compared to males (Tharenou, 2010).

#### Building preferences

In the last stage, in which preferences are built to expatriate somewhere, valence (i.e., benefits of expatriation) is found in countries where, at minimum, the individual needs can be fulfilled in a way that pushes the individual towards expatriation. As our review demonstrates, these needs are related to career, economic, political and cultural reasons. To improve valence, individuals decide to expatriate to a country that offers opportunities to address more of their individual and family needs, and actual valence can thus be better understood by analyzing it from an individual perspective. Nevertheless, our review findings provide some insight into factors that determine how valence is calculated by each type of worker. For instance, in addition to moving to a country for career, economic, political and cultural reasons, academics from developed countries appear to find additional value in expatriation if they move to a developing country where they can contribute to improving educational institutions (Lee and Kuzkabekova, 2018). Digital nomads prefer to move to a location where they can live a more convenient lifestyle (Reichenberger, 2018).

For some groups of individuals, we also identified specific needs that determine how they calculate the valence of their future expatriation. For example, non-EU individuals may pay attention to the immigration procedure in potential host countries and prefer to move to a country that offers the most simple and convenient procedure (Mielly et al., 2017). Additionally, religious individuals seem to have additional specific needs that they seek to fulfill in their future host country. For this group, *valence* is calculated by evaluating whether the location offers them the freedom to practice their religion freely or whether the country is liberal and not dominated by religious extremism, which in the latter case applies specifically to Muslim individuals moving to other Muslim countries (Alonso-Garbayo and Maben, 2009; Baruch and Forstenlechner, 2017). Furthermore, our findings reveal that some research has demonstrated the resources that individuals use to determine the location that offers the most benefits (i.e., valence); however, this research is still quite limited and focuses solely on certain employees. For instance, academics visit potential host countries to gain real-life experience (Cai and Hall, 2016), which helps them build a preference for a location. Healthcare professionals use friends or representatives of companies to obtain the necessary information about countries (Alonso-Garbayo and Maben, 2009), and managers, directors and vice presidents use colleagues’ expatriation experiences to do so (Farcas and Goncalves, 2017). Future research may wish to explore whether more resources are used by other employees to help them build preferences in this final stage of the motivation process.

*Future research suggestions.* Much more research is needed to examine the means that individuals use to build their final preferences. As individuals at this stage have already decided whether they will expatriate as SIEs or AEs (Andresen et al., 2014), we recommend that future researchers distinguish between the two types of expats when studying how they build preferences to expatriate.

### Practical Implications

Our review is useful for individuals who intend to move abroad on their own initiative. For instance, our review reveals that personal resources such as one’s personal network can be used to trigger one’s motivation to expatriate. Likewise, social media such as Facebook and LinkedIn can be used to interact with people who share similar interests about expatriation or to participate in online discussions to gain more knowledge about where one should move to address his/her individual and family needs. Our review is also useful to companies wishing to send their employees abroad for temporary assignments. Companies can influence their employees’ willingness to move abroad through their HR department. For instance, if an individual works in a company that regularly assigns employees to international postings, there must be an HR department that offers expatriation packages that include training, allowances and other benefits (Shortland, 2018). In such a company, individuals can also derive input from colleagues’ expatriation experiences (Pinto and Caldas, 2015) and thus obtain information about the value of those packages when used in practice. This information can be used by individuals who are interested in expatriation to evaluate whether they should move abroad.
